# Downregulated KIF3B Induced by miR-605-3p Inhibits the Progression of Colon Cancer via Inactivating Wnt/*β*-Catenin

**DOI:** 10.1155/2021/5046981

**Published:** 2021-08-11

**Authors:** Qilong Wang, Xiaomin Hao, Gang Xu, Tiesheng Lv

**Affiliations:** ^1^Department of General Surgery, The Third Affiliated Hospital of Xi'an Medical University, Xi'an 710068, Shaanxi Province, China; ^2^Department of Internal Medicine, Shaanxi Province Tuberculosis Hospital, Xi'an 710068, Shaanxi Province, China; ^3^Department of Oncology Surgery, The First Affiliated Hospital of Xi'an Jiaotong University, Xi'an 710068, Shaanxi Province, China

## Abstract

Colon cancer is a common malignant disease with high morbidity and mortality, and miRNA dysfunction has been confirmed as an important reason for cancer development. Several studies have verified miR-605-3p as a tumor inhibitor while its roles in colon cancer remain uncertain. In this study, the specimen of the patients and the cell lines of colon cancer were used to observe the expression of miR-605-3p, and the CCK-8, Transwell assay, and flow cytometry assay were used to observe the functions of miR-605-3p in colon cancer cells. The downstream factors of miR-605-3p were predicted by TargetScan and then were verified by dual-luciferase reporter assay. Moreover, western blot was used to investigate the effect of miR-605-3p on Wnt/*β*-catenin signal pathway. The result showed that miR-605-3p was extremely downregulated in the pathological tissues and tumor cells, and miR-605-3p could effectively induce the apoptosis and impede the proliferation and invasion of the tumor cells. It was found that KIF3B was a target of KIF3B; decreased KIF3B could reverse the effects of miR-605-3p on colon cancer. Besides, the inactivated Wnt/*β*-catenin pathway was also observed in colon cells when miR-605-3p was upregulated, and the phenomenon could be rescued by KIF3B upregulation. In conclusion, miR-605-3p could inactivate the Wnt/*β*-catenin pathway induced via promoting KIF3B expression.

## 1. Introduction

Colon cancer still remains one the most intractable diseases in the world which seriously threaten the health of human beings. Statistically, more than 1 million patients were diagnosed with colon cancer in 2018 [1, 2]. At present, drug, radiotherapy, and surgery interventions have been used to restrain the symptoms of the patients [1, 3]. However, the early symptoms of colon cancer usually do not express special natures, thus it may be mistaken as inflammation [4]. Patients' conditions have been become serious when they are first diagnosed as colon cancer [5]. Hence, even with current medical level, the prognosis and over-survival rate of the patients remain unsatisfactory. More studies are still necessary to delve the pathogenic mechanism of colon cancer. In the recent ten years, the pathological mechanism of colon cancer has received wide attention, which continually boosts the development of drug in colon cancer treatment [6, 7].

In the last decade, the functions of microRNAs in cellular activities have been confirmed by growing evidences, and the dysfunctions of miRNAs have also been observed in multiple cancers [8, 9]. miRNA dysfunction is major driving force behind the progression and recurrence [10]. Several studies have indicated that miRNA profiles in colon cancer tissues are different from normal tissues, and miRNA dysfunction has been proved as a major lead for tumor formation and development [11, 12]. Consequently, the drug therapies involved in miRNAs have been thought as prospective strategies for colon cancer treatment. miR-605-3p serves as a tumor mimic in multiple tumors while its role in colon cancer remains unclear [13].

This study aimed to provide some new target and research points for cancer development by investigating the connection of miR-605-3p and colon cancer and revealing the regulation mechanism of miR-605-3p in the progression of colon cancer.

## 2. Material and Methods

### 2.1. Specimen Preparation

This study has been approved by the hospital Ethics Committee. The tumor tissues and matched adjacent health tissues donated by the patients were used in this study. All tissues were stored at −80°C. Prior consents of the patients and authorizations from hospitals were obtained.

### 2.2. Cell Culture and Transfection

Normal human colon cell lines including FHC and HEK-293T and human hepatocellular carcinoma cell lines including HCT116, CaCO-2, SW620, and RKO were used in this study. All cells were purchased from Hunan Fenghui Biotechnology Co., Ltd. (Changsha, China). All cells were cultured with Dulbecco's modified eagle medium (DMEM, Procell Life Science and Technology Co., Ltd., China) containing 10% fetal bovine serum (FBS, Thermo Fisher, USA) at 37°C and 5% CO_2_. Subculture of the cells was performed when the cellular confluence was at 90%.

The cells were seeded into the 6-well plates, and the cell transfections were performed when the confluences of the cells were at 70%. The miR-605-3p mimics, miRNA negative control (miR-NC), pcDNA-KIF3B, and pcDNA-NC were synthesized by Generay Biotech Co., Ltd. (Shanghai, China). In short, 4 g of DNA, 100 pmol of RNA, or 10 *μ*l Lipofectamine 2000 were respectively diluted and incubated with 250 *μ*l serum-free medium for 5 min. The diluted DNA and RNA were respectively mixed with isometric diluted Lipofectamine 2000 (Thermo Fisher, Massachusetts, USA) and then were incubated at 25°C for 20 min. After that, 500 *μ*l of mixtures were added in each well, and then the cells were cultured for 24 hours.

### 2.3. Real-Time Quantitative Reverse Transcription PCR (qRT-PCR)

The miR-605-3p levels in the tissues and cell lines have been measured by qRT-PCR. The TRIzol reagent was used to perform the extractions of the total RNA in the tissues or HCC cell lines. The concentration of the total RNAs was measured by a spectrophotometer. 1 *μ*g of RNA was used to transcribe as cDNA, and a PrimeScript® RT Reagent Kit performed the cDNA transcribe with random hexamers. The reaction systems (10 *μ*L) of qRT-PCR were prepared according to the operational instruction of a KAPA qRT-PCR Kit (Sigma-Aldrich, Missouri, USA). U6 was used as the endogenous controls. The following conditions were used: denaturation at 95°C for 3 min, followed by amplification for 40 cycles at 95°C for 12 s and at 53°C for 40 s and 70°C for 30 s. The relative levels of miRNAs were calculated with the 2^−(ΔΔCt)^ method. The primers of miR-605-3p and U6 were synthesized and purified by RiboBio (Guangzhou, China). The primer sequences of miR-605-3p, KIF3B, and U6 are listed in Table 1.

### 2.4. Western Blot

The total proteins in the tissues and cell lines were extracted by RIPA buffer in an ice box, and the concentration of the extractions was measured by a BCA protein assay kit (Thermo Fisher, Massachusetts, USA). The extractions were mixed with quadruple SDS-PAGE sample loading buffer, and then the extractions were boiled at 100°C for 5 min. The proteins were transferred onto polyvinylidene fluoride (PVDF) membranes by wet transfer method. After that, the membranes were blocked with 5% fat-free milk at 4°C for 1 hour, and then the membranes were added with the related primary antibodies and incubated at 4°C overnight. The membranes were washed three times (15 min per time) by Tris buffered saline Tween (TBST), and then the membranes were incubated with second antibodies at 25°C for 1 hour. Finally, the membranes were washed three times (10 min per time) by TBST and added with ECL reagent (Thermo Fisher, USA) for observation under a chemiluminescence detection system. The antibodies purchased from Thermo Fisher (Massachusetts, USA) were used as follows: anti-KIF3B (1 : 1000), anti-*β*-catenin (1 : 1000), anti-Wnt (1 : 1000), anticleaved caspase-3 (1 : 1000), and anti-*β*-actin (1 : 1000).

### 2.5. Transwell Assay

For the invasion assay, Matrigel was diluted with eight-times DMEM, and the diluted Matrigel was added into the upper chambers of Transwells. 5 × 10^4^ cells with 200 *μ*L serum-free DMEM were seed into the upper chambers, and 600 *μ*L and DMEM containing 10% FBS were added into the cell in lower chambers. The cells were cultured for 24 hours. After that, the cell on the upper surfaces of the chambers were removed by cotton buds, and the migrated cells on the lower surface of the upper chamber were fixed by methanol for 10 min and then dried at 25°C. The cells were stained with 0.1% w/v crystal violet (Cat#G1062, Solarbio, Beijing, China) for 30 min and washed with tap water. The number of invaded cells was calculated and photographed under a Leica DMi8 microscope.

### 2.6. CCK-8 Assay

The cells (3 × 10^3^) were seeded into 96-well plates and incubated for 24 hours. The transfections were added into the related wells for further incubation. After that, the viability of the cells at 0, 24, 48, and 72 hours was measured by CCK-8 Kit (Amyjet, Wuhan, China). In short, 10 *μ*L of CCK-8 solution (Solarbio Biotechnology Co., Ltd., Shanghai, China) was added into each well, and then the cells were incubated at 25°C in dark for 4 hours. Finally, the absorbance value of each well was measured at 450 nm by a microplate reader (Molecular Devices, Shanghai, China).

### 2.7. Dual-Luciferase Reporter Gene Assay

The mutant or wild 3'-UTR sequences of FMNL2 were inserted into the pmirGLO luciferase reporter vectors (Yangjiang Bio Co., Ltd., China) to establish the FMNL2-mutant type (FMNL2-mut) and FMNL2-wild type (FMNL2-wt), respectively. FMNL2-mut and FMNL21-wt were respectively cotransfected with miR-466 mimics or miR-NC into HEK-293T cells. After that, the cells were incubated for 48 hours. Finally, the binding effect of miR-466 and FMNL2 was observed by a dual-luciferase reporter assay system.

### 2.8. Flow Cytometry Assay

HCT116 cells were harvested by trypsinase (0.25%, EDTA-free). The harvested cells were washed by 3 mL of ice phosphate-buffered saline (PBS) for once and then were fixed by alcohol. After that, 1 × 10^6^ of the cells were suspended by 100 *µ*l incubation buffer. 5 *µ*l of ice Annexin V-FITC and 5 *µ*L of propidium iodide (PI 20 *µ*g/ml) were added into the cells, and then the cells were incubated in dark for 15 min. Finally, the apoptosis level of the cells was instantly observed by a flow cytometry equipment (BD Biosciences, State of New Jersey, USA).

### 2.9. Statistical Analysis

All experiments were performed at least 3 times, independently. The data were analyzed by SPSS 20.0, and the figures were charted by GraphPad Prism 8.0. The difference of the data was tested with chi-squared test or ANOVA with Tukey's post hoc test. *P* < 0.05 means the difference of two groups is significant.

## 3. Results

### 3.1. miR-605-3p Was Extremely Downregulated in the Pathological Tissues and Tumor Cell Lines

**Figure 1:**
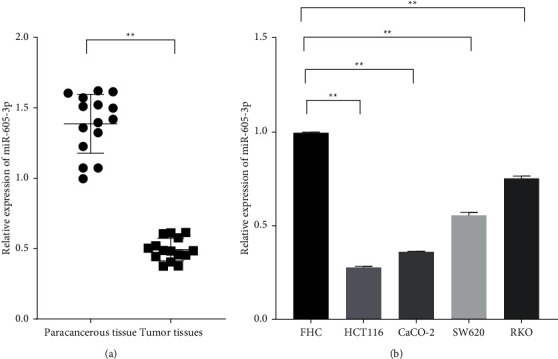
miR-605-3p was significantly downregulated in the colon cancer tissues and cell lines. (a) The relative expression levels of miR-605-3p in the paracancerous and tumor tissues of the patients with colon cancer were measured by qRT-PCR. (b) The relative expression levels of miR-605-3p in the nonneoplastic colon cells (FHC) and colon cancer cells (HCT116, CaCO-2, SW620, and RKO) were measured by qRT-PCR. ^*∗*^*P* < 0.05 and ^*∗∗*^*P* < 0.01.

The specimens of paracancerous and tumor tissues were used to confirm the difference in miR-605-3p levels. In the experiments, miR-605-3p was observably downregulated in the pathological samples compared with the normal parts (Figure 1(a); *P* < 0.01). Moreover, the decreased miR-605-3p was also observed in the colon cancer cells including HCT116, CaCO-2, SW620, and RKO compared with FHC cells (Figure 1(b); *P* < 0.01). Those observations suggested that miR-605-3p dysfunctions were related with the progression of colon cancer.

### 3.2. miR-605-3p Blocked the Progression of CaCO-2 Cells

**Figure 2:**
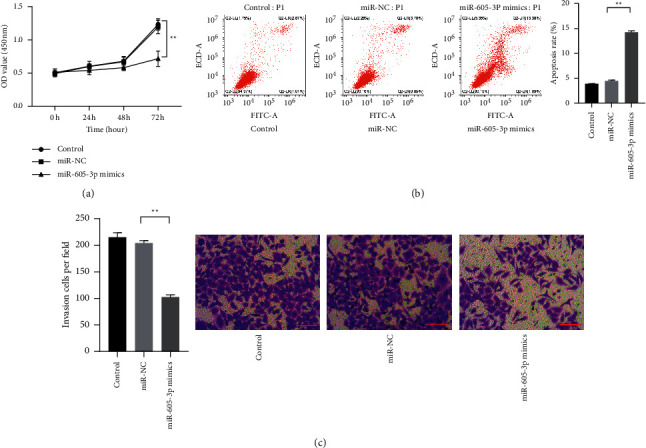
miR-605-3p inhibited the progression of colon cancer. (a) The effect of miR-605-3p on the viability of HCT116 cells was observed by CCK-8 assay. (b) The effect of miR-605-3p on the apoptosis of HCT116 cells was observed by flow cytometry assay. (c) The effect of miR-605-3p on the invasive ability of HCT116 cells was observed by Transwell assay (scale bar = 50 *μ*m). ^*∗*^*P* < 0.05 and ^*∗∗*^*P* < 0.01.

For exploring the functions of the miR-605-3p on colon cancer development, the miR-605-3p level in HCT116 cells was downregulated by the specific mimics, and the phenotypic changes in the cell growth, invasion, and apoptosis were detected by CCK-8 assay, Transwell assay, and flow cytometry assay, respectively. The results uncovered that the HCT116 cells with reduced miR-605-3p expressed low viability and invasive ability and serious apoptosis level compared to the cells without the intervention of miR-605-3p mimics (Figure 2; *P* < 0.01). Those observations suggested that declined miR-605-3p could impede the growth and invasion of HCT116 cells.

### 3.3. KIF3B Was a Target of miR-605-3p and Was Significantly Downregulated in Pathological  Tissues

**Figure 3:**
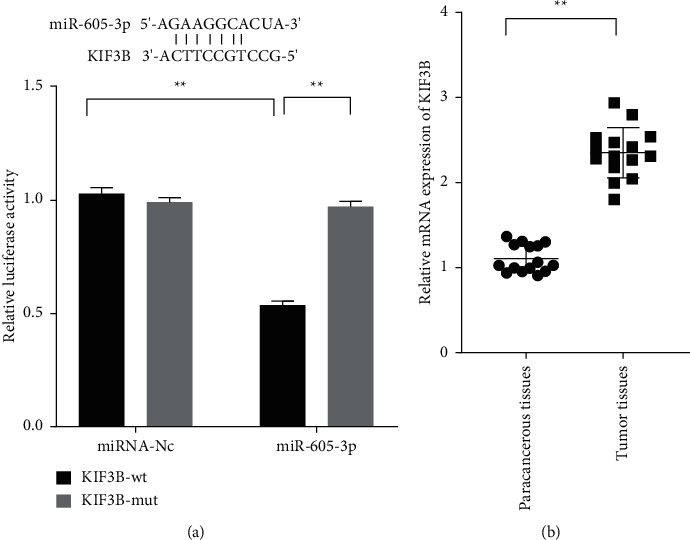
KIF3B was a target of miR-605-3p, and KIF3B was significantly upregulated in the paracancerous and tumor tissues of colon cancer. (a) The binding effect of miR-605-3p and KIF3B was measured by dual-luciferase reporter assay. (b) The relative mRNA levels of KIF3B in the specimens were measured by qRT-PCR. ^*∗*^*P* < 0.05 and ^*∗∗*^*P* < 0.01.

Given the translation barriers of miRNAs on special mRNAs, the database, TargetScan, was used to search the downstream target of miR-605-3p. It was found that miR-605-3p could directly target the 3'-UTR of KIF3B. To further verify the prediction results, the luciferase vectors containing the wild type and mutant type in the 3'-UTR sequences of KIF3B were respectively cotransfected with miR-605-3p into HEK-293T cells to observe the effect of miR-605-3p on KIF3B (Figure 3(a); *P* < 0.01). In the results, miR-605-3p expressed visible effect on wt-KIF3B rather than that on mut-KIF3B. Moreover, the increased mRNA levels of KIF3B were also found in tumor tissues (Figure 3(b); *P* < 0.01).

### 3.4. KIF3B Upregulation Reversed the Effects of miR-605-3p on Phenotype of the Cells

**Figure 4:**
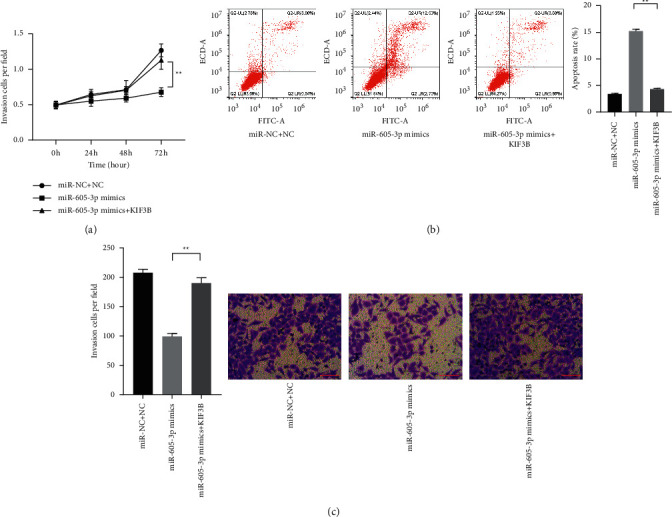
KIF3B could reverse the effects of miR-605-3p on the phenotype of colon cancer cells. (a) The effect of KIF3B on the viability of HCT116 cells with high miR-605-3p level was observed by CCK-8 assay. (b) The effect of KIF3B on the apoptosis of HCT116 cells with high miR-605-3p level was observed by flow cytometry assay. (c) The effect of KIF3B on the invasion of HCT116 cells with high miR-605-3p level was observed by Transwell assay (scale bar = 50 *μ*m). ^*∗*^*P* < 0.05 and ^*∗∗*^*P* < 0.01.

To verify whether KIF3B involves in the regulation of miR-605-3p in colon cells, the miR-605-3p mimics and KIF3B were cotransfected into the cells to observe the phenotypic changes of the cells. In the results, the reduced viability of the cells induced by miR-605-3p downregulation was reversed by KIF3B, and the cells cotransfected with miR-605-3p mimics and KIF3B expressed high invasion ability compared with the cells only transfected with miR-605-3p mimics (Figures 4(a) and 4(c); *P* < 0.01). Besides, the high apoptosis level of the cells induced by miR-605-3p was rescued by KIF3B downregulation (Figure 4(b); *P* < 0.01). Those observations suggested that the regulation of miR-605-3p in colon cancer was related with KIF3B, respectively.

### 3.5. miR-605-3p Impeded the Progression of Colon Cancer via Activating Wnt/*β*-Catenin Pathway

**Figure 5:**
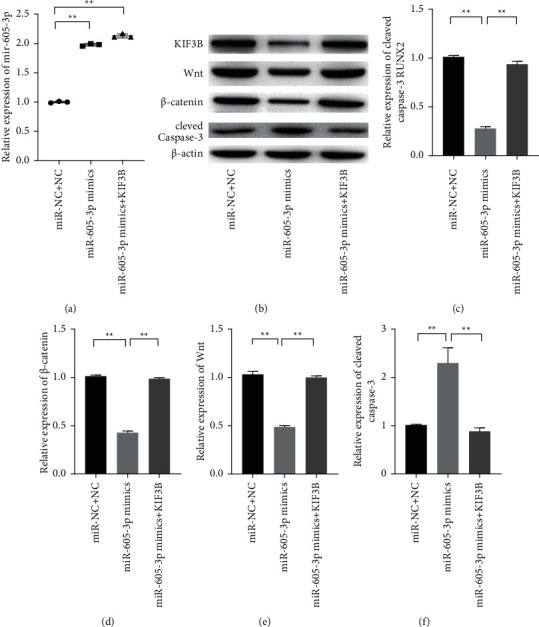
miR-605-3p inactivated the Wnt/*β*-catenin pathway via targeting KIF3B. (a) The relative expression level of miR-605-3p was measured by qRT-PCR. (b–f) The expression levels of KIF3B, Wnt, *β*-catenin, and cleaved caspase-3 were observed by western blot. ^*∗*^*P* < 0.05 and ^*∗∗*^*P* < 0.01.

To further delve the mechanism of miR-605-3p in colon cancer, the activity of Wnt/*β*-catenin pathway was observed by western blot. The results indicated that the expressions of Wnt and *β*-catenin were significantly inhibited when miR-605-3p level decreased, suggesting that the level of miR-605-3p was related with the activity of Wnt/*β*-catenin pathway (Figure 5; *P* < 0.01). However, compared with the cells singly transfected with miR-605-3p mimics, the inactivated Wnt/*β*-catenin pathway of the cells was reversed after cotransfected with KIF3B, suggesting that the tumor promotion effects of miR-605-3p were related with inactivation of Wnt/*β*-catenin pathway induced by KIF3B downregulation (Figure 5; *P* < 0.01).

## 4. Discussion

Despite recent advances in diagnosis and treatments, many patients with colon cancer still experience relapse and metastasis, primarily involving the liver, abdomen, and lung [14]. Colon cancer therefore remains an incurable disease. Previous researches have indicated that increased malignancy of CSCs serves as important reason for cancer development and resistance [15]. This study investigated the connection of miR-605-3p and colon cancer, revealed the downstream target of miR-605-3p on the progression of CSCs, and delved the regulation mechanism of miR-605-3p on colon cancer.

The profiles of miRNAs express visible difference in tumor and normal tissues of the patients, and regulation on the expression of special miRNAs has been thought as a feasible therapeutic strategy for cancer [16, 17]. For instance, one study has found that miR-34a was significantly overexpressed in colon cancer cell, and downregulated miR-34a could promote the metastasis for the cells and poor prognosis for the patients, which is analogical with the observation from the previous study [18]. This study determined that miR-605-3p was significantly downregulated in the colon cancer tissues and cell lines. The recent studies have confirmed that miR-605-3p may play a tumor support role in the diverse human tumors and involves in the malignant growth, resistance, and invasive ability [19]. In this study, it was observed that miR-605-3p inhibition could extremely inhibit the proliferation, viability, and invasive ability of CSCs.

Several studies have showed that miRNAs exert the regulation functions in cells involved in the expression of some key proteins [20]. Xi et al. have indicated that miR-204-3p upregulation counts against the growth and invasion of the colon cancer, and those effects are related with the downregulation of HMGA2 which is the downstream target of miR-204-3p [21]. Considering the natures of miRNAs on transcription restraining of proteins via targeting the 3'-UTR of the related mRNAs, the downstream targets were searched by databases including TargetScan and miRDB, and it was found that KIF3B was a downstream target of miR-605-3p. KIF3B has been confirmed to be involved in the formation and development of multiple diseases including inflammations and acute injury [22, 23]. Recently, increasing studies have indicated that aberrant KIF3B involves in the progression of some tumors [24]. Song et al. have found that KIF3B was extremely upregulated in the tissues of pancreatic cancer and related with the pTNM stage of the patients [25]. Moreover, it has been also observed that the reduced KIF3B inhibited the viability and induced the apoptosis of colon cancer [26]. In this study, KIF3B was also determined as a key factor which could visibly reverse the effects of miR-605-3p on the phenotype of the colon cancer cells. Therefore, the present proofs suggest that miR-605-3p blocked the progression of colon cancer via targeting KIF3B.

The formation and development of cancers generally involve in the activity changes of multiple signal pathways. The changes in activities of pathways in cells such as PI3K/AKT, NF-*κ*B, Hippo, and Wnt/*β*-catenin are inner force to impetus the formation and development of colon cancer [27]. The study has determined activated PI3K/AKT pathway induced by LINC00657 as a major cause leading the progression of colon cancer [28]. The related consideration has been given in the changes of the signal pathways in this study, and it was observed that the tumor cells expressed low activity of Wnt/*β*-catenin when miR-605-3p was upregulated. Moreover, KIF3B downregulation could reverse the phenomenon induced by miR-605-3p downregulation. The study has indicated that the activation of Wnt/*β*-catenin mediated by KIF3B directly promote the growth, invasion, and epithelial-mesenchymal transition of breast cancer cells [29]. Hence, this study suggests that miR-605-3p serves as a tumor inhibitor in colon cancer via suppressing the activation of Wnt/*β*-catenin induced by KIF3B.

In this study, the evidence of miR-605-3p involving in the progression of colon cancer was provided, the downstream target of miR-605-3p was determined, and the regulation mechanism of miR-605-3p in colon cancer was revealed and elaborated. However, for further verifying the practical therapeutic effects of miR-605-3p on colon cancer, the oncogenicity of miR-605-3p should be validated by animal experiments.

## Figures and Tables

**Table 1:** Primer sequences of miR-605-3p, KIF3B, and U6.

Name of primers	Sequences
miR-605-3p-F	5'-AACGAGACGACGACAGAC-3'
miR-605-3p-R	5'-AGAAGGCACTATGAGATTTAGA-3'
KIF3B-F	5'- GATGTTAAGCTGGGGCAGGT-3'
KIF3B-R	5'-TTTGCCGTCCACTAGAGCAG-3'
U6-F	5'-CTCGCTTCGGCAGCACA-3'
U6-R	5'-AACGCTTCACGAATTTGCGT-3'

## Data Availability

The datasets used and/or analyzed during the present study are available from the corresponding author on reasonable request.
